# Fungus Hæmatodes Found in the Fœtus

**Published:** 1830-01-01

**Authors:** 


### Fungus Hæmatodes in the Fœtus.

In the Journal de Progrés, tome XIV, a notice of an instance of this kind is inserted by a gentleman who signs himself, Tonnelé, D.C. Tubercles, it is well known, have been found in the fœtus in utero, but we certainly are not aware that any of the genuine malignant growths have been discovered, or recorded to have been discovered, at so early a stage of human existence.

On the 9th of December, 1827, M. Tonnelé was summoned to assist two of his confréres in conducting to its termination a protracted labour, in which the back of the child presented. On our author's arrival, he found that the feet had with difficulty been brought into the vagina, that the uterus was in a state of complete inactivity, and that the waters had been discharged a long time previously. By the joint exertions of all engaged the whole of the child, except the head, was delivered, but the uterus could not be prevailed on to contract, and the final extraction was only accomplished at last by means of the blunt hook introduced into the mouth of the fœtus, after the forceps had failed. The child was hydrocephalic, but what excited most attention was an enormous tumour of fungus hæmatodes, attached to the right parietal bone, and forming a kind of double head. The base or origin of this medullary tumour appeared to be seated in the osseous tissue of the cranium, which it perforated like a sieve; the dura mater was sound. The serum contained in the cranium might be estimated at about a pint, and the cerebral substance was soft, and macerated in appearance.

The mother of the child was thirty years of age; the father, eighty, but stouter and stronger than many men at sixty. Neither of the parents had ever laboured under any cancerous affection. We are satisfied from the description that the above was really a case of fungus hæmatodes, as we have witnessed several such tumours in adults, and in every case they had their origin in the cranial bones, more especially the diploë. As we before observed, we are not aware that medullary sarcoma has hitherto been discovered in the human infant prior to its entrance into "this piping world."

